# Understanding the Trauma of Menstrual Irregularity After COVID Vaccination: A Bird’s-Eye View of Female Immunology

**DOI:** 10.3389/fimmu.2022.906091

**Published:** 2022-06-13

**Authors:** Rinki Minakshi, Safikur Rahman, Archana Ayaggari, Durgashree Dutta, Abhishek Shankar

**Affiliations:** ^1^ Department of Microbiology, Swami Shraddhanand College, University of Delhi, New Delhi, India; ^2^ Munshi Singh College, Bhim Rao (BR) Ambedkar Bihar University, Muzaffarpur, India; ^3^ Department of Biochemistry, Jan Nayak Chaudhary Devilal Dental College, Sirsa, India; ^4^ Department of Radiation Oncology, All India Institute of Medical Sciences, Patna, India

**Keywords:** COVID-19 vaccine, menstruation, immunology, inflammation, microbiota

## Abstract

The intricacies in various signaling routes involved in the menstrual cycle can be impacted by internal as well as external stimuli, and the role of stress, be it physical, psychological, or social, in disturbing the process could be debilitating for a woman. The global endeavor of vaccination rose to protect individuals from the severity of COVID-19, but a conjunction of a short-lived menace of menstrual disturbance in the female population came out as an unsettling side effect. An understanding of the immunological panorama in the female reproductive tract (FRT) becomes important to fathom this issue. The close-knit microenvironment in the FRT shows active microbiota in the lower FRT, but the latest findings are ascertaining the presence of low-biomass microbiota in the upper FRT as well. Concerted signaling, wherein inflammation becomes an underlying phenomenon, results when a stressor elicits molecules of the inflammatory cascade. Learning lessons from the gut microbiota, we need to address the exploration of how FRT microbiota would impose inflammation by manipulating the immune response to vaccines. Since there is a prominent sex bias in the immune response to infectious diseases in women and men, the role of sex hormones and cortisol becomes important. The treatment regimen may be considered differently in women who also consider their ovarian cycle phases. Women exert robust immune response to antigenic encounters *via* cell-mediated and humoral arms. The inclusion of women in vaccine trials has been marginalized over the years, which resulted in unwanted high dosage administration of vaccines in women.

## Introduction

The complex function of regular menstruation is under the systemic endocrine control of secretions from the hypothalamus, pituitary gland, ovaries, and uterus endometrium. Methodical ovulation and hence menstruation gauge the overall wellbeing of a female body, which has been indexed as the “fifth vital sign.” The intricate chemistry of various signaling events occurring during menstruation is highly impacted by the internal as well as external stimuli, which often shows as short-lived changes in the regular pattern of menstrual periodicity and/or characteristics in every woman (like intensity and duration of bleeding as well as the accompanying pain).

The female reproductive tract (FRT) is a highly malleable signaling cascade; it can withstand stressors through the short-term manifestation of changes in the menstrual pattern. Inflammation may lead to suppression of ovarian function. The global endeavor of vaccination rose to protect individuals from the severity of COVID-19, but the conjunction of a short-lived menace of menstrual disturbance in the female population came out as an unsettling side effect.

Here in this review, we have analyzed the possible mechanisms that might be playing roles in the menstrual disturbances after COVID-19 vaccination.

## COVID-19 Vaccines and Their Impact on Menstrual Status

The unprecedented development of the COVID-19 vaccine marks itself as a savior during the days of the COVID-19 pandemic (1). Across the globe, until the date of the present study, 36 vaccines have been approved for emergency inoculation by relevant regulatory authorities in various countries (2). The most commonly used vaccines leveraged either mRNA-based (Pfizer and Moderna) or adenovirus-based (Johnson & Johnson and AstraZeneca) technology. Some of the common side effects claimed by vaccine manufacturers were fever, fatigue, headache, body ache, and swelling. However, the progression of vaccination rollouts accompanied feeds from various media articles and social media platforms that the COVID-19 vaccine was affecting the menstrual status in women, which included delay or early menstruation, heavier bleeding patterns, painful sessions, and breakthrough bleeding.

Although the change in the menstrual pattern after vaccination is not universal, its effect is quite significant. According to the UK Medicine and Healthcare products Regulatory Agency (MHRA), 39,591 suspected cases of menstrual disturbance were reported, wherein the prominent vaccine candidates were AstraZeneca, Pfizer, and Moderna (3). A study by Edelman et al., on a US cohort of 2,403 individuals, reported that 55% of the cohort receiving Pfizer vaccine, 35% relating to Moderna, and 7% linking to Johnson & Johnson/Janssen vaccine underwent a small change in cycle length (4).

In another study on the Norwegian young adult cohort, significant disturbance in the menstrual cycle was reported, which included a heavier bleeding pattern, an increase in the duration of menstruation, and a shortening of interval between two cycles (5).

Although the study by Edelman et al. identified small changes in cycle length, which might not be of concern with respect to the clinical range of 8 days or more (change in cycle length), the data from such studies are still valuable for individual planning or avoiding pregnancy. The same study recorded 358 women who were vaccinated with both doses in the same cycle, and interestingly this group had 10.6% of women who experienced a change in the length of their menstrual cycle for more than 8 days. However, the cycle length in all the groups under study returned to normal after two cycles (4).

## Incidences From Past Vaccinations

The administration of prophylactic vaccines for human papillomavirus (HPV) is currently under use for preventing cervical cancer (6). However, there are reports of premature ovarian insufficiency (POI) after HPV vaccination. POI is a condition wherein there is a failure in ovarian function showing amenorrhea with the underlying pathophysiology of higher gonadotrophins and lower estradiol (7). Gong et al. have reported increased follicle-stimulating hormone (FSH), amenorrhea, menstrual irregularities, and premature menopause associated with the HPV vaccine (6). Reports studying feasible pathogenesis behind this association highlighted the aggravation of autoimmune response (8). In the first report of its kind, menstrual abnormalities were observed in Japanese women after inoculation against hepatitis B (9). A similar disturbance was reported in 1913 after typhoid vaccination (10).

## Immunology of Menstruation

The immunological panorama of the uterus keeps changing with the menstrual cycle. The mucosa of the FRT displays a plethora of immune activity. The uterus and fallopian tubes, which are in the upper reproductive tract, sporadically become exposed to a burst of various antigens like sperm or fetal-placental organization, whereas the lower reproductive tract comprising the vagina and cervix harbors a niche of the microbiome that altogether represents a different antigenic stimulus. The multiple layers of the squamous epithelial lining of the lower reproductive tract lack tight junctions, whereas the upper single-layered epithelium has tightly bound columnar cells. This is important, as the transition zone between the two epithelia acts as a major effector and site of induction for cell-mediated immunity {reviewed in (11)}. Contrasting the respiratory and gastrointestinal mucosae, the FRT displays IgG circulation {reviewed in (11)}.

### The Innate Immune Panorama

The pattern recognition receptors (PRRs) rise as the first line of defense in the vaginal tract. The toll-like receptors (TLRs) expressed on uterine epithelia elicit inflammatory response {reviewed in (12)}. There is a whole array of specific TLR mRNA and changes in protein expression during the span of the menstrual cycle {reviewed in (12)}. There is evidence that NOD-like receptors (NLRs) and TLRs have been known to induce the expression of cytokine cascade (13, 14). Studies have shown that the interaction between host and microbiota is done through PRRs (12).

Neutrophils, macrophages, and dendritic cells (DCs) are highly distributed in the upper FRT. Activation of memory T cells by macrophages and DCs acts as a bridge between innate and adaptive responses. Before ovulation, innate lymphoid cells (ILCs) are low in number, but they increase swiftly after ovulation. The immune reaction in the lower FRT keeps shifting during the menstrual cycle, increasing susceptibility toward infection {reviewed in (15)}.

**Figure 1:**
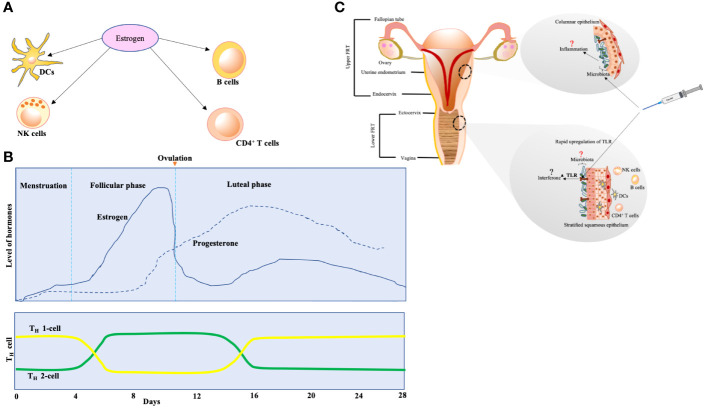
**(A)** The effect of estrogen on various sentinels of innate immunity. The expression of estrogen receptors is activated on dendritic cells (DCs), natural killer (NK) cells, CD4^+^ T cells, and B cells. The differentiation of DCs from bone marrow is stimulated, and secretion of cytokines and chemokines by immature DCs is prompted under the effect of estrogen. B cells are encouraged to produce IgM and IgG because of estrogen. The cytotoxicity of NK cells is reduced by estrogen. **(B)** T-cell population variation according to estrogen and progesterone levels over the span of various phases of 28-day menstrual cycle. The effect on T cells is biphasic—higher estrogen levels (seen during follicular phase of menstrual cycle) encourage polarization of TH-2 cells (these cells actively participate in allergic reactions and drive B-cell class switching for IgE production that arms the effector cells, mast cells, and basophils), whereas lower estrogen levels (seen during luteal phase and menstruation) prompt differentiation of TH-1 cells (these are critical in clearance of invading pathogens but exaggerated stimulus associates with autoimmune diseases). **(C)** The potential effect of vaccination on female reproductive tract (FRT). Upper FRT: endometrium seems to dock a relatively unique microbiota with lesser biomass (19). The microbiota may be modulating the events of inflammation in endometrium. Lower FRT: the vaccine might be eliciting rapid upregulation of TLR in the lower FRT mucosa, which could potentially lead to interferon activation. This mechanism was seen in yellow fever virus vaccine, that led women to respond strongly as compared to men {reviewed in (20)}. This might be one of the reasons behind adverse events after vaccination in women.

The potential connection shared between the immune system and sex hormones was proposed by Calzoari (16). The immunocompetence of women is recorded not only with higher levels of CD4^+^ T cells and immunoglobulins (Ig) but also with more incidences of autoimmune disorders {reviewed in (17)}. Receptors for estrogen are widely expressed on DCs, macrophages, natural killer cells, T cells, and B cells, which indicates the role played by estrogen in immunocompetence regulation {reviewed in (18)}. The number of CD4^+^ T cells is higher in women as compared to men. **Figure 1A** illustrates the immune panorama in the FRT.

Estrogen and progesterone levels vary during the phases of the menstrual cycle, and estrogen affects the chemokine receptors in CD4^+^ T cells, which have important consequences for T-cell homing (**Figure 1B**) during infection and autoimmunity {reviewed in (18)}.

Fluctuations in levels of hormones during various phases of the menstrual cycle can have an impression on the immune response in the mucosa. In studies on stress biomarkers, C-reactive protein (CRP), a sentinel of the acute innate response, has been associated with fluctuations in ovarian hormones during changes in the phases of the menstrual cycle (21). The integral role of cytokines and chemokines in the development of inflammation is absolute. In cases of smallpox vaccine, the effect of cytokines was emphasized on not only the efficacy of vaccine but also events of side effects {reviewed in (22)}.

## Response to Infectious Diseases Is Indeed Sex-Biased

Although the immune response in women to infectious diseases like viral infection is robustly juxtaposed with that in men, the incidences of autoimmune disorders are higher in women {reviewed in (18)}. Summing up the results from various studies, the better response to infection in women is caused by the higher levels of estrogen that curtails pro-inflammatory cytokine expression. Instances of viral infections like the Hantavirus and SARS-CoV-1 (which caused an outbreak in 2003) have disclosed that women have a more robust immune response than men {reviewed in (18)}. The Institute of Medicine recommends the assessment of incidences pertaining to different responses to infections in men and women (23).

The clinical implication of sex bias toward infectious studies is much needed where a detailed consideration on the account of drug pharmacokinetics in men and women should be addressed. Women mounted frequent and severe adverse reactions to HIV antiretroviral therapy {reviewed in (18)}. The effect of sex difference on the humoral response to a number of vaccines has been studied {reviewed in (24)}.

## Uterine Microbiota: What Immunology Has to Say

The long-thought paradigm about a sterile uterus has been well challenged by reports of bacterial colonization in the uterine environment (12, 25). The concept of commensal microbiota in the endometrium and placenta is growing {reviewed in (12)}. Bacterial signatures were detected in the middle endometrium of 60% of women undergoing hysterectomy (26). Microbial communities have been associated with commensalism in various parts of the human body like the skin, lung, gut, and epithelial surface. The members of these microbial communities may modulate the response of the host to various infections and hence vaccination {reviewed in (27)}. Benner et al. have reviewed the evidence on the existence of the microbiome in the endometrium and placenta (12). The concept of “microbial endocrinology” is budding wherein microbes sense changes in host hormones as their environment changes, resulting in alterations in their gene profile {reviewed in (28)}. A meta-transcriptomic analysis hypothesized that the oral microbiome was able to perceive alterations in concentrations of the stress hormone, cortisol, which resulted in metabolic changes in microbes (28). It has been studied that a short-term stress exposure impacts the gut microbiota, which subsequently influences the response to imposed stress relevant to anxiety and inflammation {reviewed in (29)}.

An astonishing concept of gender bias, termed “microgenderome,” was suggested wherein the commensal microbiota exercised gender bias by regulating sex hormones (30). The concept highlights that the structure of the commensal microbiota diverges at the time of puberty, resulting in male and female commensalism.

### Immunology of Uterine Microbiota

The impact of bacterial colonization definitely affects the immune cells of the uterine mucosa. For successful commensalism, the uterine mucosa needs to protect itself against any potential tissue invasion, which forms the basis of suggested immunological barriers seen across the homeostasis in the intestinal microbiome. As described by Hooper and Macpherson, three immunological barriers act to establish the equilibrium: the exposure of the microbial community to immune cells is limited, mediators of immune cascade keep a check on the direct contact between microbe and epithelium, and highly coordinated detection and destruction of breaching microbes (31). The endometrial mucosa suffices its potential for all three parameters (12).

The uterine leukocyte contains 10%–20% of macrophages (MΦ) and DCs, which constitute the antigen-presenting cells (APCs). The late secretory phase of the menstrual cycle shows mature APCs and MΦ along with NK cells as major producers of cytokines in the endometrium. T cells are residents of the deep endometrial layer {reviewed in (12)}. The stromal cells of healthy endometrium constitutively release CCL2 chemokine {reviewed in (12)}.

The DCs are important when it comes to introducing antigens of vaccine to T cells. The plethora of immune reactions controlled by PRRs has been shown to be modulated by microbiota, which directly affects DCs. This was shown well in the case of the intranasal inactive cholera toxin vaccine. The TLR-mediated antigen sensing by DCs in the lung lining led to the activation of gut-homing receptors, whereas the production of IgA in a TLR-dependent way was diminished in cases where microbiota was depleted due to broad-spectrum antibiotics {reviewed in (32)}. The significance of the gut microbiota in the modulation of T- and B-cell response to the vaccine has been reviewed (32).

The microbiota can strikingly impose itself as a biological adjuvant on the response of vaccine {reviewed in (32)}. The commensals have been shown to modulate the presentation of antigens by the epithelial cells of the intestine {reviewed in (32)}. The gut microbiota has enzymes (hydroxysteroid dehydrogenase (HSD)) that metabolize sex steroid hormones, and therefore alteration in the gut microbiota dramatically affects hormone metabolism, leading to immunity (20).

Microbiota might be a key factor in modulating uterus immunology (**Figure 1C**). These data on the indispensable role of microbiota (lungs, gut, etc.) should be applied to the FRT also.

## An Orchestration of Hormones: What Goes Wrong

The interaction among the brain, ovaries, and uterine mucosa is responsible for the entire process of menstruation. The role of these organs in menstrual disturbance is elaborated subsequently.

### Brain Controlling Reproductive Hormones

The brain controls the menstrual cycle by concerting cyclic induction of gonadotropin-releasing hormone (GnRH) by the hypothalamus, which further stimulates the pituitary to release FSH and luteinizing hormone (LH). The ovaries produce sex-steroid hormones, estrogen, and progesterone, which control the development of follicles, maturation, and release of oocytes under the control of FSH and LH.

### Cortisol

**Figure 2:**
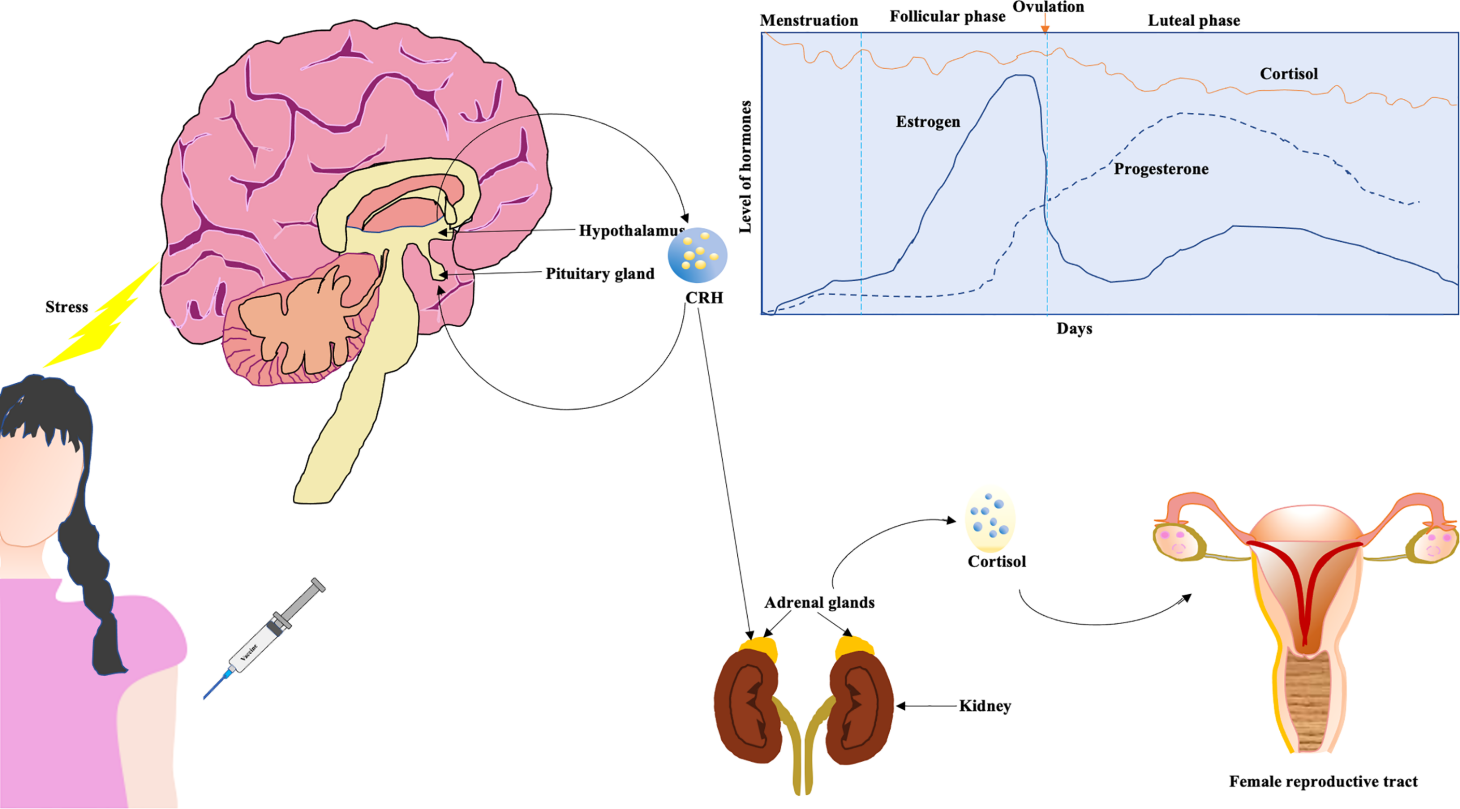
Cortisol affecting female reproductive tract. The hypothalamus-pituitary-adrenal (HPA) axis might be affected due to the imposed stress of vaccination (a higher dose). The cortisol affects female reproductive tract. Generally, circulating levels of cortisol are higher during follicular phase in contrast to luteal phase (graphical representation) (34).

The hypothalamus-pituitary-adrenal (HPA) axis controls the production as well as secretion of cortisol hormones in the adrenal cortex through corticotropin-releasing hormone (CRH). Cortisol, being a steroid hormone, is essentially a glucocorticoid that affects reproductive organs as well as response to stress, inflammation, and immune functions {reviewed in (33)}. Under stress, a cascade of hormonal and physiological reactions are displayed wherein one aspect involves stimulation of the hypothalamus by the amygdala in the brain to release cortisol from the adrenal cortex (**Figure 2**). The body’s homeostasis is maintained by cortisol, and in women, the interaction between sex hormones and cortisol is known to be an analytical determinant of stress progression and the body’s response. One meta-analysis showed that cortisol levels are higher in the follicular phase vis-à-vis the luteal phase of the menstrual cycle (34). Higher levels of cortisol potentially overwhelm normal levels of female sex hormones (35). The CRH exerts inflammatory activities after getting secreted in peripheral sites of inflammation, and there are receptors of CRH in most of the female reproductive organs that play a significant role in inflammatory components like ovulation and luteolysis (35). CRH inhibits GnRH, and cortisol suppresses LH, estrogen, and progesterone, causing resistance in the target tissues of these female sex hormones (35). So under stress, the HPA axis results in abnormalities in menstruation. Pro-inflammatory cytokines like interleukin (IL)-1β increase in the central nervous system (CNS) in reaction to cortisol-associated acute stress {reviewed in (36)}. It has been previously reported that a rise in serum corticosterone levels (in mice) corresponds to an increase in the trafficking of immune cells (37).

## Immune Response Shows Sex Bias

A plethora of studies now testify that men and women are affected differently by diseases {reviewed in (18)}. Women show lesser severity toward viral, bacterial, fungal, or parasitic infections than men, but the prevalence of sexually transmitted diseases and autoimmune disorders is higher in women. This gender discretion has been attributed to X-chromosome inactivation, differences in expression, regulation of hormones, etc. Thus, the “one drug” treatment protocol comes as a solution while addressing therapeutic interventions where sex-based disease outcome is studied {reviewed in (18)}. The treatment regimen may be different among women during their ovarian cycle phases. Women exert robust immune response to antigenic encounters *via* cell-mediated and humoral arms. Markle et al. provided remarkable insights into the immune response due to the effect of sex on the transcriptome and microbiome. The environmental factors can affect the way sex hormones act during an immune response (20).

The inclusion of women in vaccine trials has been marginalized, which resulted in unwanted high dosage administration of vaccines in women. Women in the age range of 18–64 years were seen to generate vigorously antibodies against the influenza vaccine; moreover, their reaction to a half dose of the vaccine was equivalent to the reaction of men who received a full dose {reviewed in (20)}. Despite the adverse immune response generated due to vaccination that has been evaluated in previous reports, the current vaccine trial against SARS-CoV-2 did not consider sex bias. The latest research reports that women showed 1.9 times higher side effects after COVID-19 vaccination than men (38). The report states that 10% of Israeli women experienced menstrual irregularities after receiving the third dose of the COVID-19 vaccine and concluded that women’s doses of the vaccine should be lesser than men’s (38). Alvergne et al. reported that in a sample in the United Kingdom, 20% of individuals experienced menstrual disturbances (39). The authors have emphasized that menstrual irregularity bears a close association with inflammation reaction rather than an ovulation-related issue. Additionally, the timing of vaccination also matters in female subjects.

## Is Vaccine Constitution a Stressor?

Vaccines with mRNA and adenovirus-vector bases have been shown to elicit higher levels of CD4^+^ and CD8^+^ T-cell response to SARS-CoV-2 (40). Studies discussed in this review show that women receiving both mRNA vaccines (Pfizer and Moderna) and viral vector-based vaccines (Johnson &Johnson and AstraZeneca) underwent menstrual disturbance.

Edelman et al. have proposed that the robust immune reaction stimulated by an mRNA vaccine could be a potential stressor affecting the hypothalamus-pituitary-ovarian axis (4). They have reported that the variation in cycle length could be due to the imposed stress (vaccination timing) during the follicular phase of the menstrual cycle.

## Conclusion

Stress is any physical or psychological tension that threatens the homeostasis of an organism. The situation of the COVID-19 pandemic exposed individuals to psychological stress, and the decision to undergo vaccination against the SARS-CoV-2 was a part of it. The news pouring in about menstrual irregularities after vaccination stemmed from the willingness of women to be vaccinated (41, 42). The reaction to vaccination is different from person to person.

The stimulation of the HPA axis under stress results in cortisol elevation, which exerts a global effect on body functioning. Hitherto, studies on the effect of stress resulting in dysbiosis majorly includes majorly the gut (43) and vaginal microbiome (44), which focused on the impaired immune system. However, research on the effect of stress on the uterine microbiome is still in its infancy. Although 16S rRNA-based studies on commensals of the FRT (26) are well-established, there are still different views regarding uterine microbiota. One important observation was made on the phenomenon behind the discretionary power of host epithelium on commensal and pathogenic bacteria that the adherent commensals do not activate TLRs whereas pathogenic bacteria with virulence factors like flagellin breach the epithelial barrier, leading to TLR activation {reviewed in (20)}. Studies like these can be replicated in uterine microbiota to ascertain whether the uterine microbiota originates from contamination/infection or if it is indigenous.

As per our discussion elsewhere, the oral microbiome showed the effects of microbial endocrinology. This phenomenon is applied in FRT microbiome studies and might help in ascertaining the outcomes of infections as well as vaccination. Therefore, a comprehensive elucidation of the effect of stress on uterine dysbiosis is warranted.

It is now undebatable that COVID-19 vaccination has protected against the severity of the disease. The world population grappled with the problems caused by the infection of SARS-CoV-2, and being vaccinated boosted our confidence to face this situation. However, the issue of menstrual irregularities in the female population after vaccination is something that needs attention. The issue of postvaccination menstrual disturbance is medically very important with respect to female wellbeing because an unexpected event causes anxiety. We must design studies acknowledging the vulnerable groups like those with autoimmune disorders or preexisting gynecological issues so that they can be counseled appropriately before vaccination. An editorial by Victoria Male has highlighted one misinformation being disseminated to the public that the COVID-19 vaccine leads to infertility in women (45). So the issue of menstrual disturbance after vaccination would exaggerate such misinterpretations. Hence, a comprehensive study on this issue needs scientific attention in view of public awareness. Although one article talked about this problem as being short-lived (46), still we need more studies to explore this dimension so that women are mentally prepared for the postvaccination menstrual side effects.

## Limitations

Since the advent of vaccines, not only humans but also animals and plants have benefitted. The female population deserves to be considered with respect to their biological constitution in vaccine trials. The literature on issues like this is scarce. Hence it is high time to encourage female-specific vaccine research to address subjects like menstrual disturbance, which, even though short-lived, impacts women physically, emotionally, and socially.

## Author Contributions

RM contributed to the conception and design of the study. SR, AA, DD, and AS contributed to the revision and read and approved the submitted version of the manuscript. All authors listed have made a substantial, direct, and intellectual contribution to the work and approved it for publication.

## Author Disclaimer

Some citations are from non-peer-reviewed websites. Therefore, expert discretion is advised.

## Conflict of Interest

The authors declare that the research was conducted in the absence of any commercial or financial relationships that could be construed as a potential conflict of interest.

## Publisher’s Note

All claims expressed in this article are solely those of the authors and do not necessarily represent those of their affiliated organizations, or those of the publisher, the editors and the reviewers. Any product that may be evaluated in this article, or claim that may be made by its manufacturer, is not guaranteed or endorsed by the publisher.
